# Expression of Lamin A gene in periodontitis: A cross-sectional study

**DOI:** 10.34172/japid.2025.003

**Published:** 2025-01-20

**Authors:** Naser Sargolzaie, Jalil Tavakkol-Afshari, Raziyeh Mohebati, Faeze Basiri, Arsalan Shahri, Mahdiye Fasihi Ramandi

**Affiliations:** ^1^Dental Research Center, School of Dentistry, Mashhad University of Medical Science, Mashhad, Iran; ^2^Immunology Research Center, BuAli Research Institute, Mashhad University of Medical Sciences, Mashhad, Iran; ^3^Dental Materials Research Center, Mashhad Dental School, Mashhad University of Medical Sciences, Mashhad, Iran; ^4^Department of Periodontics, School of Dentistry, Shahid Beheshti University of Medical Sciences, Tehran, Iran

**Keywords:** Biomarkers, Electrophoresis, Lamin A, PCR, Periodontitis

## Abstract

**Background.:**

The host defense process against invading bacteria leads to the destruction of the periodontium. Lamin A is an important protein for protecting DNA and preventing premature cell aging. This study investigated the expression of the Lamin A gene in periodontitis patients.

**Methods.:**

Using an analytical cross-sectional design, Lamin A gene expression was evaluated in 23 periodontitis patients and 24 healthy individuals referred to the Periodontology Department of Mashhad Dental School and Hekmat Clinic, Mashhad, Iran. Gingival samples were collected, followed by RNA extraction, cDNA synthesis, and real-time PCR. Statistical analyses were conducted using SPSS.

**Results.:**

While the age distribution did not show significant differences between the groups, gender distribution was statistically different. Therefore, the study focuses on comparing Lamin A gene expression levels between the patient and healthy groups, separated by gender. Considering the analysis of 47 gingival tissue samples, the Lamin A gene expression level was higher in healthy participants, with the difference being statistically significant only in female participants (198.45±54.00 in healthy females vs. 143.52±29.29 in periodontitis females).

**Conclusion.:**

These findings suggest that the expression of the Lamin A gene was higher in healthy individuals than in periodontitis patients. More studies are needed to draw more accurate conclusions. If confirmed in larger studies, this protein group might serve as potential biomarkers, enhancing periodontitis assessment strategies.

## Introduction

 Periodontal disease is a common infectious condition originating from biofilms and can ultimately lead to the loss of periodontal supportive structures.^1^ The invasion of biofilms into the gingival sulcus triggers an immune response, leading to gingivitis and periodontitis.^2^ If the inflammation persists unaddressed, the disease progresses, culminating in the loss of periodontal structures, a condition referred to as periodontitis.^3^ The balance between the host immune response and bacterial load is influenced by several factors, including immune deficiencies and increased bacterial burden, leading to greater tissue damage and progression of periodontitis. Certain virulent bacteria, including those in the red complex, are involved in the occurrence of periodontal disease.^4^

 Disease severity tends to be higher in smokers.^5^ Due to impaired wound healing processes, the incidence and severity of the disease are also elevated in diabetic patients.^6^ Gingival crevicular fluid (GCF), found in the gingival sulcus and periodontal pocket, contains both bacterial and host secretory proteins. Various proteins within GCF play critical roles in periodontal disease, highlighting the need to identify periodontal biomarkers to support disease diagnosis and progression monitoring.^7^ Lamin A is an intranuclear protein that helps protect DNA and prevent premature cell aging. This protein forms a filamentous network within the nuclear envelope’s inner structure. Proteins from this family are involved in chromatin domain organization, DNA transcription, translation, cell aging, and nuclear strength. The Lamin A gene codes for Lamin A and Lamin C, two proteins with slightly different amino acid sequences. Lamin A undergoes multiple synthesis stages and is two exons longer than Lamin C, which is synthesized directly.^8^ Other members of this family include Lamin B1 and Lamin B2, with Lamin A typically found in well-differentiated tissues and Lamin B in low-differentiated tissues and cells. Mutations in the Lamin gene can result in a wide range of hereditary diseases.^9^

 Oral diseases, including dental caries, periodontal diseases, and oral cancer, significantly contribute to the global burden of chronic diseases. Poor oral health diminishes quality of life and increases the risk of other chronic diseases.^10^ Therefore, identifying predictive or preventative factors is crucial for enhancing overall health. This study investigated the expression of the Lamin A gene in periodontal disease, focusing on differences between periodontal patients and healthy individuals.

## Methods

 This analytical cross-sectional study was approved by the Ethics Committee of Mashhad University of Medical Sciences (Approval ID: IR.MUMS.DENTISTRY.REC.1400.042). This study examined the expression level of the Lamin A gene in two groups: 24 healthy individuals and 23 periodontitis patients. Samples were collected from individuals at the Periodontology Department of Mashhad Dental School and Hekmat Clinic in Mashhad, Iran.

 Inclusion criteria for healthy individuals included patients requiring crown lengthening surgery without signs of gingivitis, periodontitis, or bone loss. The periodontitis group’s inclusion criteria included individuals with bleeding on probing (BOP), alveolar bone loss, and pocket depths > 5 mm, who required flap surgery.

 After surgery, a gingival sample of at least 1 × 3 mm was collected from either the marginal or attached gingiva. These gingival samples were carefully placed in microtubes containing RNAlater to preserve their integrity. The samples were stored at 4 °C and transported to the Bu-Ali Research Institute (Mashhad, Iran) within 24 hours. Gingival tissue RNA was extracted using the Total RNA Extraction Kit (Parstous, Iran) following the manufacturer’s instructions. To ensure the stability of the mRNA, it was converted to cDNA using the Easy cDNA Synthesis Kit (Parstous, Iran). To prevent contamination, the samples and equipment were sterilized using UV. Primers for real-time PCR were synthesized per Metabion Kit (Sinuhebiotech, Iran) specifications; Table 1 presents the sequences.

 Lamin A gene expression was evaluated using the SYBR Green real-time polymerase reverse transcription PCR (RT-PCR) technique. Primers were designed using Beacon Designer software (version 7.9) and NCBI Primer online software and ordered from Pishgam Company, Tehran, Iran (https://www.pishgambc.com/). Primer specificity and reaction products were confirmed by sequencing through Bioneer Company, South Korea. Real-time PCR was performed on a LightCycler 96 System.

 Using GAPDH (glyceraldehyde-3-phosphate dehydrogenase) as the housekeeping gene, real-time RT-PCR amplification conditions were as follows: initial denaturation at 95 °C for 10 minutes, followed by 40 cycles of denaturation at 95 °C for 10 seconds, annealing at 60 °C for 30 seconds, and extension at 72 °C for 20 seconds. The reaction was performed in a total volume of 10 μL, including 0.4 μL forward primer (10 pmol/μL), 0.4 μL reverse primer (10 pmol/μL), 0.2 μL distilled water, 5 μL master mix, and 4 μL cDNA.

 Gene expression data were analyzed using the 2^ΔΔCt^ method, with GAPDH chosen as the housekeeping gene.

 Data analysis was conducted using SPSS 12 (SPSS Inc., Chicago, IL, USA), with statistical tests including the Mann-Whitney test, paired t-test, independent t-test, one-way ANOVA, and Shapiro-Wilk test for data distribution. The results were considered statistically significant at* P* < 0.05.

## Results

 Forty-seven gingival tissue samples from 24 healthy individuals and 23 periodontitis patients were examined, including 30 women and 17 men, with a mean age of 37.36 ± 11.88 years. The Shapiro-Wilk test confirmed a normal distribution for all quantitative variables (*P* > 0.05).

 There was no significant age difference between groups (*P* = 0.638, Table 2); however, a significant gender difference was observed between groups (*P* = 0.004) using the chi-squared test. Therefore, subsequent analyses focused on comparing age and Lamin A gene expression levels between patient and healthy groups, stratified by gender.

**Figure 1:**
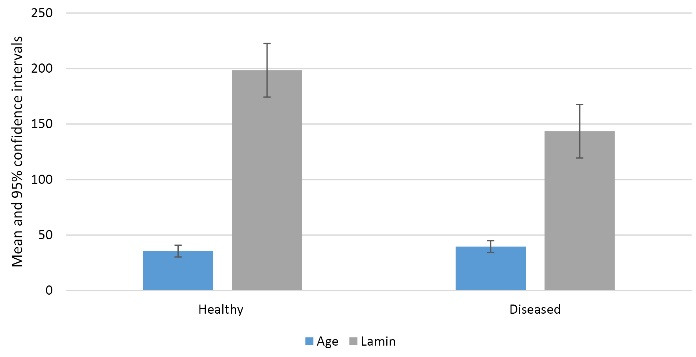
Mean and 95% confidence interval of variables by groups in women

 Lamin A gene expression was significantly lower in periodontitis patients compared to healthy individuals (*P* = 0.006, Table 3). Among male participants, the mean age and Lamin A gene expression were higher in the healthy group than in the periodontitis group, although this difference was not statistically significant (*P* = 0.069, Table 3, Figure 1).

**Table 1:** Sequence of primers for SYBR Green real-time polymerase chain reaction assay

**Primer name**	**Sequence**
Lamin forward	5'-AGC AAA GTG CGT GAG GAG TT-3'
Lamin reverse	5'-AGG TCA CCC TCC TTCTTG GT-3’
GAPDH-forward	5'-CCCATCACCATCTTCCAGG-3'
GAPDH-reverse	5'-CATCACGCCACAGTTTCCC-3’

GAPDH: Glyceraldehyde-3-phosphate dehydrogenase

**Table 2:** Group comparison in terms of age and gender

**Group**	**Size**	**Age (Mean±SD)**	**Gender N (%)**
Healthy individuals	24	36.46 ± 13.17	Female	20 (83.3%)
			Male	4 (16.7%)
Periodontitis patients	23	38.30 ± 10.58	Female	10 (43.5%)
			Male	13 (56.5%)
*P* value		* Z = 0.47 *P* = 0.638	**χ2 = 8.08 *P* = 0.004

* Mann-Whitney U test results. **Chi-squared test.

**Table 3:** Comparison of age and Lamin A expression between groups, split by gender

**Gender**	**Variable**	**Group**	**Number**	**Mean±SD**	**Independent T-test**
Female	Age	Healthy	20	35.65 ± 11.81	T = 0.86 *P* = 0.395
		Patient	10	39.70 ± 12.71	
	Lamin A expression	Healthy	20	198.45 ± 54.00	T = 2.99 *P* = 0.006
		Patient	10	143.52 ± 29.29	
Male	Age	Healthy	4	40.50 ± 20.49	T = 0.12 *P* = 0.956
		Patient	13	37.23 ± 9.02	
	Lamin A expression	Healthy	4	184.06 ± 19.49	T = 1.96 *P* = 0.069
		Patient	13	157.59 ± 24.61	

## Discussion

 As periodontal disease is widespread and the second most common oral disease,^11^ many researchers are actively seeking specific factors to identify susceptible individuals. A specific biomarker should have the characteristics of disease diagnosis, indicating disease severity, treatment response, and prognosis.^12^

 Kornman et al^13^ observed that interleukin-1 (IL-1) coding gene pleomorphism increases the disease severity in periodontitis patients. In a study by Gibert et al,^14^ the level of alkaline phosphatase enzyme in chronic periodontitis patients had an inverse association with the amount of bone loss. In another study, an increase in IL-3, IL-4, IL-5, blood albumin, alpha-amylase, and a decrease in cystatin-1 levels were reported in patients with chronic periodontitis.^15^ Many identified biomarkers also increase in other inflammatory conditions.^16^ Additionally, these biomarkers may be linked to infection or collagen and bone destruction, which often manifest later in disease progression.^17^ Hence, the critical point is to find an appropriate specific biomarker. Currently, no studies have investigated Lamin A as a biomarker in periodontitis. Lamin glycoprotein is one of the main components of the basement membrane and plays a role in DNA protection and cell aging processes.^18^ Lamin A increases during periodontal ligament development.^19^It has been demonstrated that the amount of Lamin protein expression by periodontal fibroblasts affects the chemical response created by gingival epithelial cells.^8^

 Increased expression of Lamin A/C has been observed in the apical migration of gingival epithelial cells.^20^ This study showed that the Lamin A expression gene in healthy individuals was higher than in periodontitis patients.

 According to Alhudiri et al,^21^ high expression of Lamin A/C was correlated with a good prognosis of breast cancer. The lowest expression of Lamin A/C can be seen in low differentiation tumors, with a high probability of metastasis and inadequate inflammatory response around the tumor. Another study showed that the Lamin A expression directly affects the apoptosis cycle and cell division.^22^ It seems that Lamin A has a protective role in lining tissues and plays a vital role in the repair, growth, and differentiation of PDL. According to the results of this study, the expression rate of Lamin A was higher in healthy individuals. This result was seen among women but was not significant in men. Lower Lamin A gene expression may correlate with poorer prognosis and increased disease severity, as observed in healthy and periodontitis groups among women but not men.

 One of the limitations we encountered in this study was the small number of healthy male participants. The small sample size of healthy males limits generalizability and may reduce statistical power to detect significant effects. Therefore, it is recommended that a larger sample size be considered in future studies. This study is among the first to examine Lamin A gene expression levels in periodontal disease. Further studies are needed to confirm these findings, potentially allowing Lamin A to serve as a predictor of periodontitis treatment outcomes. To deepen our understanding, future research should explore the mechanistic role of Lamin A in periodontal pathogenesis. Longitudinal studies are recommended to validate its prognostic implications, offering insights into how Lamin A expression influences treatment outcomes.

## Conclusion

 The higher expression of the Lamin A gene in healthy individuals compared to periodontitis patients suggests that reduced Lamin A expression during inflammatory processes may be linked to disease severity and tissue destruction.

## Competing Interests

 The authors declare no conflict(s) of interest related to the publication of this work.

## Consent for Publication

 Not applicable.

## Data Availability Statement

 The data that support the findings of this study are available on request from the corresponding author.

## Ethical Approval

 This study was approved by the Ethics Committee of Mashhad University of Medical Sciences,

 Mashhad, Iran (IR.MUMS.DENTISTRY.REC.1400.042).

## References

[1] Pihlstrom BL, Michalowicz BS, Johnson NW (2005). Periodontal diseases. Lancet.

[2] Moeintaghavi A, Arab HR, Amiri Moghaddam M, Shahmohammadi R, Yalood Bardan B, Soroush Z (2019). Evaluation of effect of surgical and nonsurgical periodontal therapy on serum C-reactive protein, triglyceride, cholesterol, serum lipoproteins and fasting blood sugar in patients with severe chronic periodontitis. Open Dent J.

[3] Papapanou PN, Sanz M, Buduneli N, Dietrich T, Feres M, Fine DH (2018). Periodontitis: consensus report of workgroup 2 of the 2017 World Workshop on the Classification of Periodontal and Peri-Implant Diseases and Conditions. J Periodontol.

[4] Socransky SS, Haffajee AD, Cugini MA, Smith C, Kent RL Jr (1998). Microbial complexes in subgingival plaque. J Clin Periodontol.

[5] Bergström J (2004). Tobacco smoking and chronic destructive periodontal disease. Odontology.

[6] Nazir MA (2017). Prevalence of periodontal disease, its association with systemic diseases and prevention. Int J Health Sci (Qassim).

[7] Tsuchida S, Satoh M, Kawashima Y, Sogawa K, Kado S, Sawai S (2013). Application of quantitative proteomic analysis using tandem mass tags for discovery and identification of novel biomarkers in periodontal disease. Proteomics.

[8] Ohshima M, Yamaguchi Y, Otsuka K, Sato M, Ishikawa M (2006). Laminin expression by human periodontal ligament fibroblasts. Connect Tissue Res.

[9] Stick R, Peter A (2020). Evolution of the lamin protein family at the base of the vertebrate lineage. Cell Tissue Res.

[10] Kazemian A, Hoseinzadeh M, Banihashem Rad SA, Jouya A, Tahani B (2023). Nudging oral habits; application of behavioral economics in oral health promotion: a critical review. Front Public Health.

[11] Otenio CC, Fonseca I, Martins MF, Ribeiro LC, Assis NM, Ferreira AP (2012). Expression of IL-1β, IL-6, TNF-α, and iNOS in pregnant women with periodontal disease. Genet Mol Res.

[12] Ji S, Choi Y (2015). Point-of-care diagnosis of periodontitis using saliva: technically feasible but still a challenge. Front Cell Infect Microbiol.

[13] Kornman KS, Crane A, Wang HY, di Giovine FS, Newman MG, Pirk FW (1997). The interleukin-1 genotype as a severity factor in adult periodontal disease. J Clin Periodontol.

[14] Gibert P, Tramini P, Sieso V, Piva MT (2003). Alkaline phosphatase isozyme activity in serum from patients with chronic periodontitis. J Periodontal Res.

[15] da Rós Gonçalves L, Soares MR, Nogueira FC, Garcia C, Camisasca DR, Domont G (2010). Comparative proteomic analysis of whole saliva from chronic periodontitis patients. J Proteomics.

[16] Loos BG, Van Dyke TE (2020). The role of inflammation and genetics in periodontal disease. Periodontol 2000.

[17] Seymour GJ, Gemmell E (2001). Cytokines in periodontal disease: where to from here?. Acta Odontol Scand.

[18] Ohshima M, Tokunaga K, Sato S, Maeno M, Otsuka K (2003). Laminin- and fibronectin-like molecules produced by periodontal ligament fibroblasts under serum-free culture are potent chemoattractants for gingival epithelial cells. J Periodontal Res.

[19] Denes BJ, Bolton C, Illsley CS, Kok WL, Walker JV, Poetsch A (2019). Notch coordinates periodontal ligament maturation through regulating lamin A. J Dent Res.

[20] Terranova VP, Lyall RM (1986). Chemotaxis of human gingival epithelial cells to laminin. A mechanism for epithelial cell apical migration. J Periodontol.

[21] Alhudiri IM, Nolan CC, Ellis IO, Elzagheid A, Rakha EA, Green AR (2019). Expression of lamin A/C in early-stage breast cancer and its prognostic value. Breast Cancer Res Treat.

[22] Kaspi E, Frankel D, Guinde J, Perrin S, Laroumagne S, Robaglia-Schlupp A (2017). Low lamin A expression in lung adenocarcinoma cells from pleural effusions is a pejorative factor associated with high number of metastatic sites and poor Performance status. PLoS One.

